# Synthesis, crystal structure and properties of the trigonal–bipyramidal complex tris­(2-methyl­pyridine *N*-oxide-κ*O*)bis­(thio­cyanato-κ*N*)cobalt(II)

**DOI:** 10.1107/S2056989024003050

**Published:** 2024-04-11

**Authors:** Christian Näther, Inke Jess

**Affiliations:** a Institut für Anorganische Chemie, Universität Kiel, Germany; Tokyo University of Science, Japan

**Keywords:** synthesis, crystal structure, discrete complex, trigonal–bipyramidal coordination, thermal properties, cobalt thio­cyanate, 2-methyl­pyridine *N*-oxide

## Abstract

Reaction of Co(NCS)_2_ with 2-methyl­pyridine *N*-oxide in a 1:3 ratio in *n*-butanol leads to the formation of crystals of tris­(2-methyl­pyridine *N*-oxide-κ*O*)bis­(thio­cyanato-κ*N*)cobalt(II), [Co(NCS)_2_(C_6_H_7_NO)_3_]. The asymmetric unit of the title compound consists of one Co^II^ cation two thio­cyanate anions and three crystallographically independent 2-methyl­pyridine *N*-oxide coligands in general positions. The Co^II^ cations are trigonal–bipyramidally coordinated by two terminal N-bonding thio­cyanate anions in the *trans*-positions and three 2-methyl­pyridine *N*-oxide coligands into discrete complexes. These complexes are linked by inter­molecular C–H⋯S inter­actions into double chains that elongate in the *c*-axis direction. Powder X-ray diffraction (PXRD) measurements prove that all batches are always contaminated with an additional and unknown crystalline phase. Thermogravimetry and differential analysis of crystals selected by hand reveal that the title compound decomposes at about 229°C in an exothermic reaction. At about 113°C a small endothermic signal is observed that, according to differential scanning calorimetry (DSC) measurements, is irreversible. PXRD measurements of the residue prove that a poorly crystalline and unknown phase has formed and thermomicroscopy indicates that some phase transition occurs that is accompanied with a color change of the title compound.

## Abstract 2

In the crystal structure of the title compound, discrete complexes with a rare Co coordination are found, in which the Co^II^ cations are fivefold coordinated by two thio­cyanate anions and three 4-methyl­pyridine *N*-oxide coligands within a slightly distorted trigonal–bipyramidal coordination.

## 1. Chemical context

Coordination compounds based on Co^II^ are an inter­esting class of compounds, for example, in the field of mol­ecular magnetism. They can form discrete complexes, which are promising compounds as single-mol­ecule or single-ion magnets (Böhme *et al.*, 2018 ▸; Buchholz *et al.*, 2012 ▸; Ziegenbalg *et al.*, 2016 ▸). If the Co^II^ cations are linked by small-sized ligands into networks, single-chain magnetism might be observed (Ceglarska *et al.*, 2021 ▸; Mautner *et al.*, 2018*a* ▸; Rams *et al.*, 2017 ▸, 2020 ▸). This is the case for example for compounds in which the cations are linked by pairs of thio­cyanate anions into chains and this is one reason why we have been inter­ested in this class of compounds for several years.

In the course of these investigations we have prepared a large number of compounds with pyridine derivatives as coligands in which the thio­cyanate anions are either only terminally N-bonded or act as μ-1,3-bridging ligands. The former coordination mostly leads to the formation of discrete complexes, in which the cobalt cations shows an octa­hedral coordination. With very strong donor coligands, in a few cases discrete tetra­hedral complexes are observed (Mautner *et al.*, 2018*a* ▸; Neumann *et al.*, 2018 ▸), whereas compounds with a fivefold coordination are very rare. This is also obvious from a search in the CSD, which confirms this trend (Näther & Jess, 2024*a* ▸). In this regard, it is noted that we have reported the first Co(NCS)_2_ chain compound, in which the cobalt cations show an alternating five- and sixfold coordination (Böhme *et al.*, 2022 ▸).

In further work we also used O-donor coligands such as pyridine *N*-oxide derivatives (Näther & Jess, 2023 ▸). With 4-methyl­pyridine *N*-oxide we obtained two different discrete complexes with the composition Co(NCS)_2_(4-methyl­pyridine *N*-oxide)_4_ and Co(NCS)_2_(4-methyl­pyridine *N*-oxide)_3_, of which the first compound shows the usual octa­hedral coord­ination, whereas the second compound exhibits a trigonal–bipyramidal coordination (Näther & Jess, 2024*a* ▸). Surprisingly, the complex with a fivefold coordination can easily be prepared, whereas for the octa­hedral complex only a very few crystals were accidentally obtained, indicating that the compound with a fivefold coordination is more stable.

In this context, the question arises as to whether this observation can be traced back to the nature of the coligand. Therefore, a search in the CSD was performed, which revealed that only eleven Co(NCS)_2_ compounds with pyridine *N*-oxide derivatives and related ligands have been reported that always show an octa­hedral coordination. Nevertheless we tried to prepare new compounds with 2-methyl­pyridine *N*-oxide, which is similar to 4-methyl­pyridine *N*-oxide used in previous work. Within these investigations we obtained a compound with the composition Co(NCS)_2_(2-methyl­pyridine *N*-oxide) in which the Co^II^ cations are octa­hedrally coordinated and linked into layers by μ-1,3(*N*,*S*)-bridging thio­cyanate anions and μ-1,1(*O*,*O*)-bridging 2-methyl­pyridine *N*-oxide coligands (Näther & Jess, 2024*b* ▸). Later, we additionally obtained a further compound that was characterized by single crystal X-ray diffraction. This proved that a discrete complex with the composition Co(NCS)_2_(2-methyl­pyridine *N*-oxide)_3_ had been obtained in which the Co^II^ cations show a trigonal–pyramidal coordination, as was the case with 4-methyl­pyridine *N*-oxide as coligand. 
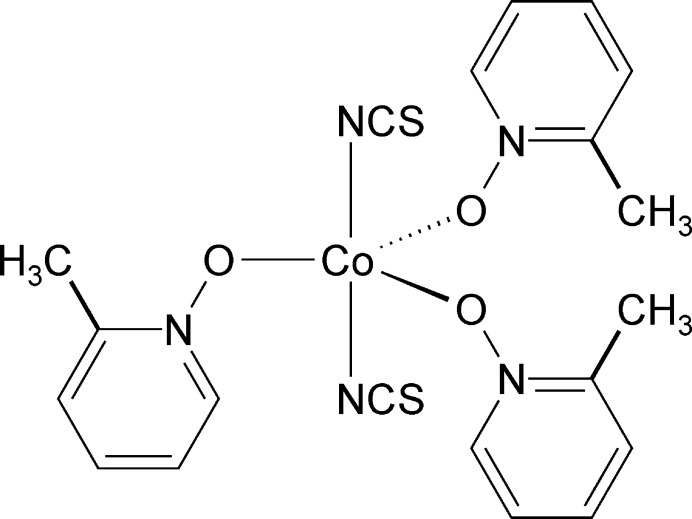


## 2. Structural commentary

**Figure 1:**
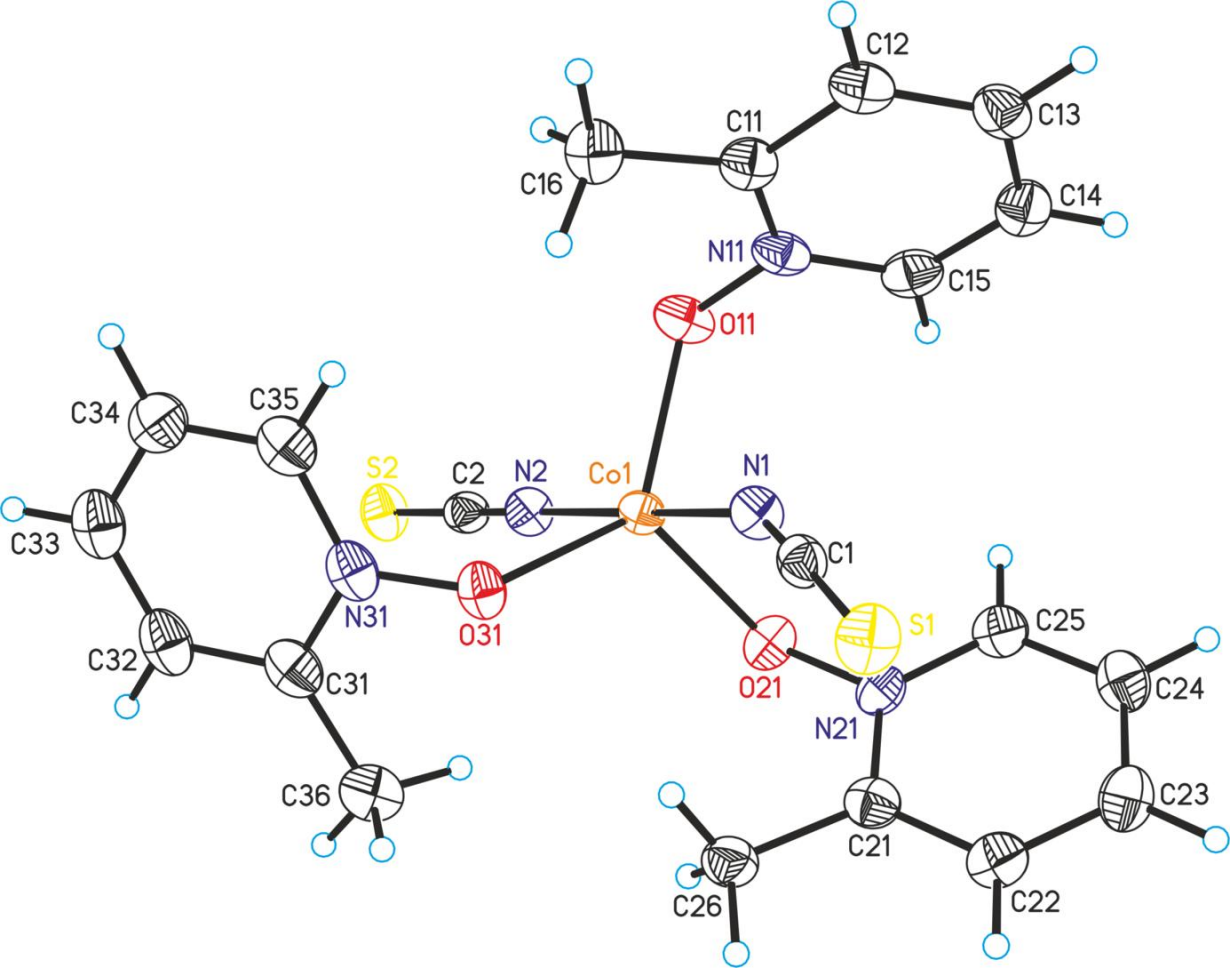
Crystal structure of the title compound with labeling and displacement ellipsoids drawn at the 50% probability level.

The asymmetric unit consists of one Co^II^ cation, two crystallographically unique thio­cyanate anions and three distinct 2-methyl­pyridine *N*-oxide coligands, all located in general positions (Fig. 1 ▸). In the crystal structure, the cobalt cations are fivefold coordinated by two terminally N-bonded thio­cyanate anions and three 2-methyl­pyridine *N*-oxide coligands into discrete complexes. From the bond angles it is obvious that a slightly distorted trigonal–bipyramidal coordination is formed (Table 1 ▸) with the 2-methyl­pyridine *N*-oxide coligands in the equatorial and the anionic ligands in the axial position, as is the case for Co(NCS)_2_(4-methyl­pyridine *N*-oxide)_3_ already reported in the literature (Näther & Jess, 2024*a* ▸). Therefore, the bond lengths and angles of the title compound are similar to those in the 4-methyl­pyridine *N*-oxide compound.

In this context, it is noted that the Co(NCS)_2_ compound with only 2-methyl­pyridine forms discrete tetra­hedral complexes, which presumably can be traced back to steric crowding because the methyl group is adjacent to the coordinating N atom (Refcode DEYGAR; Wöhlert *et al.*, 2013 ▸). With 3-methyl­pyridine, two discrete complexes with the composition Co(NCS)_2_(3-methyl­pyridine)_2_ (Refcode EYARIG; Boeckmann *et al.*, 2011 ▸) and Co(NCS)_2_(3-methyl­pyridine)_4_ (Refcodes EYAROM and EYAROM01; Boeckmann *et al.*, 2011 ▸ and Małecki *et al.*, 2012 ▸) have been reported, of which the first shows a tetra­hedral, whereas the second an octa­hedral coordination. Finally, discrete complexes are also known with 4-methyl­pyridine, including several solvates, in which the Co^II^ cations always show an octa­hedral coordination [Refcodes VERNUC (Harris *et al.*, 2003 ▸), CECCOC (Micu-Semeniuc *et al.*, 1983 ▸), XIHHEB and XIHHEB01 (Harris *et al.*, 2001 ▸, 2003 ▸)].

## 3. Supra­molecular features

**Figure 2:**
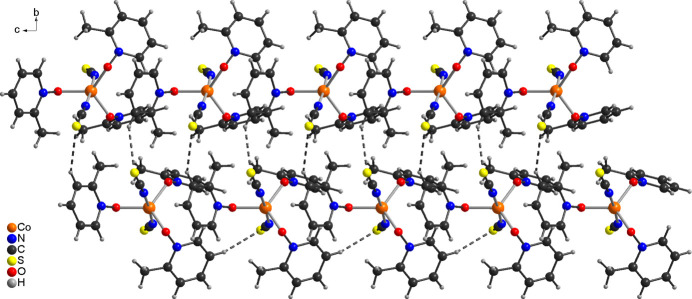
Crystal structure of the title compound with a view down *a* of part of a double chain. Inter­molecular C—H⋯S contacts are shown as dashed lines.

**Figure 3:**
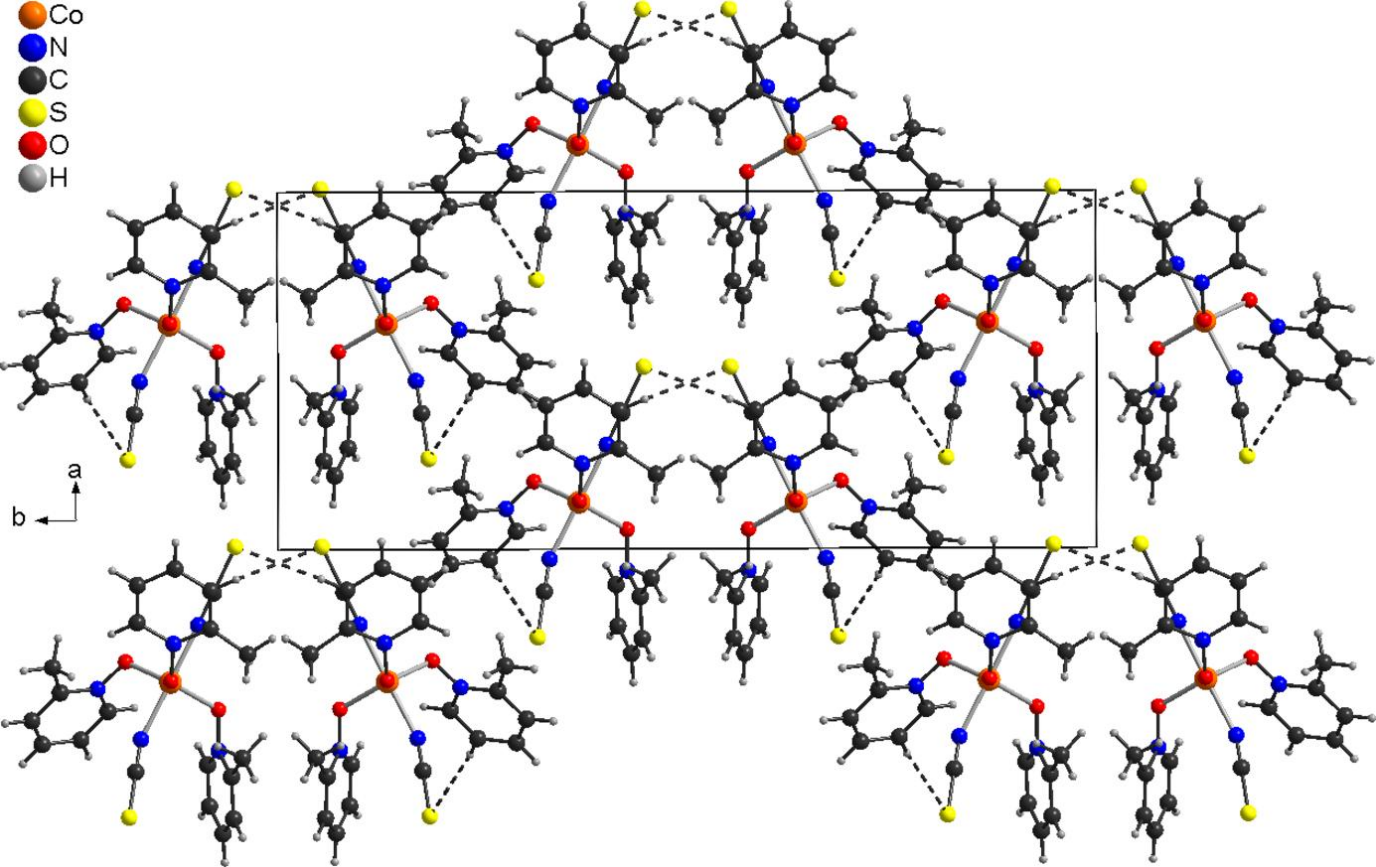
Crystal structure of the title compound with a view along the crystallographic *c*-axis direction. Inter­molecular C—H⋯S contacts are shown as dashed lines.

In the crystal structure of the title compound, the discrete complexes are linked by inter­molecular C—H⋯S hydrogen bonding into chains for which one contact with an C–H⋯S angle close to linearity is responsible (Fig. 2 ▸ and Table 2 ▸). The chains are joined by an additional much weaker C—H⋯S contact into double chains that elongate along the *c*-axis direction (Figs. 2 ▸ and 3 ▸ and Table 2 ▸). From Fig. 3 ▸, the non-centrosymmetric arrangement of the complexes becomes obvious. Finally, there is one intra­chain and one inter­chain C—H⋯O contact, but from the H⋯O distances and the C—H⋯O angles, they only correspond to weak inter­actions (Table 2 ▸).

## 4. Database survey

A search in the CSD (version 5.43, last update March 2023; Groom *et al.*, 2016 ▸) using CONQUEST (Bruno *et al.*, 2002 ▸) reveal that no Co(NCS)_2_ compounds with 2-methyl­pyridine *N*-oxide have been reported. If the search is expanded to all transition metals, three hits are found, including a discrete Zn complex with a fivefold coordination with the composition Zn(NCS)_2_(2-methyl­pyridine *N*-oxide)_2_(H_2_O) (Refcode UKIMEI; Mautner *et al.*, 2016 ▸) and a polymeric Cd compound with an octa­hedral coordination with the composition Cd(NCS)_2_(2-methyl­pyridine *N*-oxide) (Refcode UKILIL; Mautner *et al.*, 2016 ▸). Finally, there is one compound with the composition Mn(NCS)_2_(2-methyl­pyridine), which is isotypic to the Cd compound mentioned before (Mautner *et al.*, 2018*b* ▸).

If one searches for cobalt thio­cyanate compounds with pyridine *N*-oxide derivatives, eleven structures are found in which the cobalt cations are always octa­hedrally coordinated. In most structures, the thio­cyanate anions are only terminally N-bonded, which leads to the formation of mononuclear or dinuclear complexes or compounds with chain structures [Refcodes FONBIU (Shi *et al.*, 2005 ▸), IDOYEG (Shi *et al.*, 2006*a* ▸), VAZDAB (Craig *et al.*, 1989 ▸), FATJAN (Cao *et al.*, 2012 ▸) and FATJER (Cao *et al.*, 2012 ▸)].

Finally, there are also compounds with chain or layered structure with pyridine *N*-oxide derivatives that contain μ-1,3-bridging thio­cyanate anions [TILHIG (Shi *et al.*, 2007 ▸), REKBUF (Shi *et al.*, 2006*b* ▸), TERRAK (Zhang *et al.*, 2006*a* ▸), MEQKOJ (Zhang *et al.*, 2006*b* ▸), UMAVAF (Zhang *et al.*, 2003 ▸) and UMAVUZ (Zhang *et al.*, 2003 ▸)].

## 5. Additional investigations

Based on single-crystal data measured at room temperature, a powder pattern was calculated and compared with the experimental pattern, which reveals that the title compound is contaminated with an additional and unknown phase (Fig. S1). Several batches with different ratios between Co(NCS)_2_ and 2-methyl­pyridine *N*-oxide in different solvents were prepared, but it was not possible to obtain the title compound as a pure phase.

**Figure 4:**
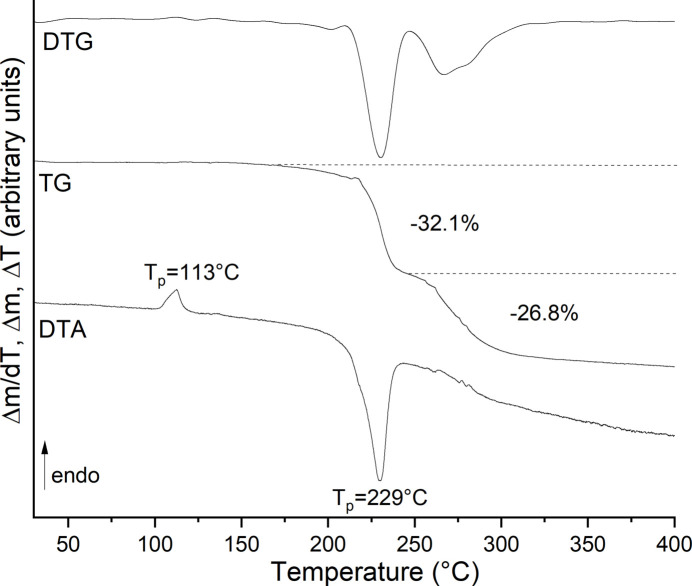
DTG, TG and DTG curves for the title compound. The mass loss is given in % and the peak temperature in °C. For this measurement, crystals selected by hand were used.

Nevertheless, the thermal properties of the title compound were investigated by thermogravimetry and differential thermoanalysis (TG-DTA) measurements using crystals separated by hand. Upon heating, two mass losses were observed that according to the DTG curve are poorly resolved and that are accompanied with a strong exothermic event in the DTA curve, indicating the decomposition of the coligands (Fig. 4 ▸). There is one weak endothermic signal at about 113°C, where the sample mass does not change. Measurements using differential scanning calorimetry (DSC) and PXRD prove that this event is irreversible and leads to a new phase of very poor crystallinity (Figs. S2 and S3). Thermomicroscopic investigations reveal that this process is accompanied with a change of the color of this compound and that it leads to a destruction of the single crystals, indicating that a reconstructive phase transition occurred (Fig. S4).

## 6. Synthesis and crystallization

**Synthesis**

Co(NCS)_2_ (99%) and 2-methyl­pyridine *N*-oxide (96%) were purchased from Sigma Aldrich and *n*-butanol (99.5%) from Carl Roth.

Single crystals of the title compound were obtained by the reaction of 0.5 mmol (87.4 mg) of Co(SCN)_2_ and 1.5 mmol (163.7 mg) of 2-methyl­pyridine *N*-oxide in 1 mL of *n*-butanol. Within 3 d, crystals suitable for structure analysis were obtained together with some powder of an unknown crystalline phase. We also tried other solvents such as methanol or ethanol and we varied the ratio between Ni(NCS)_2_ and 2-methyl­pyridine but the title compound was never obtained as a pure phase.

**Experimental details**

X-ray powder patterns were measured using a Stoe Transmission Powder Diffraction System (STADI P) equipped with a linear, position-sensitive MYTHEN 1K detector from Stoe & Cie and a XtaLAB Synergy, Dualflex, HyPix diffractometer from Rigaku, both with Cu *K*α radiation.

Thermogravimetry and differential thermoanalysis (TG-DTA) measurements were performed in a dynamic nitro­gen atmosphere in Al_2_O_3_ crucibles at 8°C min^−1^ using a STA-PT 1000 thermobalance from Linseis. The DSC measurements were performed using a DSC 1 Star System with STARe Excellence Software from Mettler-Toledo AG at 10°C min^−1^. Thermomicroscopy was performed with a hot-stage from Linkam and a microscope from Olympus. All thermoanalytical instruments were calibrated using standard reference materials.

## 7. Refinement

Crystal data, data collection and structure refinement details are summarized in Table 3 ▸. The hydrogen atoms were positioned with idealized geometry (methyl H atoms allowed to rotate but not to tip) and were refined with *U*_ĩso_(H) = 1.2*U*_eq_(C) (1.5 for methyl H atoms) using a riding model. The absolute structure was determined and is in agreement with the selected setting.

## Supplementary Material

Crystal structure: contains datablock(s) I. DOI: 10.1107/S2056989024003050/jp2003sup1.cif

Structure factors: contains datablock(s) I. DOI: 10.1107/S2056989024003050/jp2003Isup2.hkl

Figure S1. Two experimental powder pattern of the residues obtained by reacting Co(NCS)2 with 2-methylpyridine in ratio 1:3 in n-butanol (A and B) together with the powder pattern of the title compound calculated from single crystal data obtained at room-temperature (C). DOI: 10.1107/S2056989024003050/jp2003sup3.png

Figure_S2. DSC heating and cooling curve for the title compound. DOI: 10.1107/S2056989024003050/jp2003sup4.png

Figure S3. Experimental powder pattern of the residue obtained after the DSC measurement (top) and powder pattern of the title compound calculated from single crystal data obtained at room-temperature. DOI: 10.1107/S2056989024003050/jp2003sup5.png

Figure S4. Microscopic images of crystals of the title compound at different temperatures. DOI: 10.1107/S2056989024003050/jp2003sup6.png

CCDC reference: 2347581

Additional supporting information:  crystallographic information; 3D view; checkCIF report

## Figures and Tables

**Table 1:** Selected geometric parameters (Å, °)

Co1—N1	2.086 (3)	Co1—O21	2.016 (2)
Co1—N2	2.051 (3)	Co1—O31	2.037 (3)
Co1—O11	1.992 (3)		
			
N2—Co1—N1	176.35 (13)	O21—Co1—N2	91.70 (12)
O11—Co1—N1	91.87 (12)	O21—Co1—O31	118.20 (11)
O11—Co1—N2	88.87 (12)	O31—Co1—N1	84.17 (12)
O11—Co1—O21	114.88 (12)	O31—Co1—N2	92.52 (12)
O11—Co1—O31	126.83 (11)	C1—N1—Co1	155.7 (3)
O21—Co1—N1	91.23 (11)		

**Table 2:** Hydrogen-bond geometry (Å, °)

*D*—H⋯*A*	*D*—H	H⋯*A*	*D*⋯*A*	*D*—H⋯*A*
C14—H14⋯S1^i^	0.95	2.95	3.868 (4)	163
C15—H15⋯O31^i^	0.95	2.47	3.296 (5)	145
C32—H32⋯S2^ii^	0.95	3.02	3.873 (4)	150
C26—H26*B*⋯O31	0.98	2.64	3.512 (5)	149

Symmetry codes: (i) 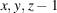 ; (ii) 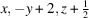 .

**Table 3:** Experimental details

Crystal data	
Chemical formula	[Co(NCS)_2_(C_6_H_7_NO)_3_]
*M*_r_	502.47
Crystal system, space group	Monoclinic, *C* *c*
Temperature (K)	100
*a*, *b*, *c* (Å)	11.9020 (2), 26.4007 (2), 7.1933 (1)
β (°)	104.299 (1)
*V* (Å^3^)	2190.26 (5)
*Z*	4
Radiation type	Cu *K*α
μ (mm^−1^)	8.21
Crystal size (mm)	0.2 × 0.05 × 0.05

Data collection	
Diffractometer	XtaLAB Synergy, Dualflex, HyPix
Absorption correction	Multi-scan (*CrysAlis PRO*; Rigaku OD, 2022 ▸)
*T*_min_, *T*_max_	0.581, 1.000
No. of measured, independent and observed [*I* > 2σ(*I*)] reflections	24478, 4183, 4137
*R*_int_	0.036
(sin θ/λ)_max_ (Å^−1^)	0.639

Refinement	
*R*[*F* ^2^ > 2σ(*F* ^2^)], *wR*(*F* ^2^), *S*	0.033, 0.089, 1.04
No. of reflections	4183
No. of parameters	283
No. of restraints	2
H-atom treatment	H-atom parameters constrained
Δρ_max_, Δρ_min_ (e Å^−3^)	0.62, −0.28
Absolute structure	Flack *x* determined using 1732 quotients [(*I* ^+^)−(*I* ^−^)]/[(*I* ^+^)+(*I* ^−^)] (Parsons *et al.*, 2013 ▸)
Absolute structure parameter	−0.003 (3)

Computer programs: *CrysAlis PRO* (Rigaku OD, 2022 ▸), *SHELXT2014/4* (Sheldrick, 2015*a* ▸), *SHELXL2016/6* (Sheldrick, 2015*b* ▸), *DIAMOND* (Brandenburg & Putz, 1999 ▸), *XP* in *SHELXTL-PC* (Sheldrick, 2008 ▸) and *publCIF* (Westrip, 2010 ▸).

## supplementary crystallographic information

### Crystal data

**Table:**

[Co(NCS)_2_(C_6_H_7_NO)_3_]	*F*(000) = 1036
*M**_r_* = 502.47	*D*_x_ = 1.524 Mg m^−^^3^
Monoclinic, *C**c*	Cu *K*α radiation, λ = 1.54184 Å
*a* = 11.9020 (2) Å	Cell parameters from 17140 reflections
*b* = 26.4007 (2) Å	θ = 3.4–79.0°
*c* = 7.1933 (1) Å	µ = 8.21 mm^−^^1^
β = 104.299 (1)°	*T* = 100 K
*V* = 2190.26 (5) Å^3^	Needle, pink
*Z* = 4	0.2 × 0.05 × 0.05 mm

### Data collection

**Table:**

XtaLAB Synergy, Dualflex, HyPix diffractometer	4183 independent reflections
Radiation source: micro-focus sealed X-ray tube, PhotonJet (Cu) X-ray Source	4137 reflections with *I* > 2σ(*I*)
Mirror monochromator	*R*_int_ = 0.036
Detector resolution: 10.0000 pixels mm^-1^	θ_max_ = 80.2°, θ_min_ = 3.4°
ω scans	*h* = −14→15
Absorption correction: multi-scan (CrysAlisPro; Rigaku OD, 2022)	*k* = −33→33
*T*_min_ = 0.581, *T*_max_ = 1.000	*l* = −9→8
24478 measured reflections	

### Refinement

**Table:**

Refinement on *F*^2^	Hydrogen site location: inferred from neighbouring sites
Least-squares matrix: full	H-atom parameters constrained
*R*[*F*^2^ > 2σ(*F*^2^)] = 0.033	*w* = 1/[σ^2^(*F*_o_^2^) + (0.0636*P*)^2^ + 1.9394*P*] where *P* = (*F*_o_^2^ + 2*F*_c_^2^)/3
*wR*(*F*^2^) = 0.089	(Δ/σ)_max_ = 0.002
*S* = 1.04	Δρ_max_ = 0.62 e Å^−^^3^
4183 reflections	Δρ_min_ = −0.28 e Å^−^^3^
283 parameters	Absolute structure: Flack *x* determined using 1732 quotients [(*I*^+^)-(*I*^-^)]/[(*I*^+^)+(*I*^-^)] (Parsons *et al.*, 2013)
2 restraints	Absolute structure parameter: −0.003 (3)
Primary atom site location: dual	

### Special details

**Table:**

Geometry. All esds (except the esd in the dihedral angle between two l.s. planes) are estimated using the full covariance matrix. The cell esds are taken into account individually in the estimation of esds in distances, angles and torsion angles; correlations between esds in cell parameters are only used when they are defined by crystal symmetry. An approximate (isotropic) treatment of cell esds is used for estimating esds involving l.s. planes.

### Fractional atomic coordinates and isotropic or equivalent isotropic displacement parameters (Å^2^)

**Table:**

	*x*	*y*	*z*	*U*_iso_*/*U*_eq_	
Co1	0.63119 (5)	0.86737 (2)	0.79970 (6)	0.01961 (14)	
N1	0.4719 (3)	0.83113 (11)	0.7698 (4)	0.0235 (6)	
C1	0.3811 (3)	0.82456 (12)	0.7982 (5)	0.0227 (7)	
S1	0.25390 (8)	0.81632 (4)	0.84239 (13)	0.0304 (2)	
N2	0.7907 (3)	0.90130 (11)	0.8467 (4)	0.0243 (6)	
C2	0.8812 (3)	0.92031 (13)	0.8804 (5)	0.0233 (7)	
S2	1.00934 (8)	0.94704 (4)	0.92598 (13)	0.0313 (2)	
O11	0.6887 (2)	0.81252 (9)	0.6570 (4)	0.0277 (6)	
N11	0.6138 (3)	0.77822 (11)	0.5616 (5)	0.0250 (6)	
C11	0.6035 (3)	0.73262 (14)	0.6406 (6)	0.0260 (7)	
C12	0.5254 (3)	0.69782 (14)	0.5330 (6)	0.0269 (7)	
H12	0.518236	0.665128	0.584075	0.032*	
C13	0.4589 (3)	0.71024 (15)	0.3547 (6)	0.0288 (8)	
H13	0.404785	0.686632	0.283627	0.035*	
C14	0.4715 (3)	0.75755 (15)	0.2791 (6)	0.0292 (8)	
H14	0.425779	0.766777	0.155868	0.035*	
C15	0.5502 (4)	0.79080 (14)	0.3837 (6)	0.0284 (8)	
H15	0.560585	0.823010	0.331396	0.034*	
O21	0.5544 (2)	0.92584 (9)	0.6365 (4)	0.0268 (5)	
N21	0.4395 (3)	0.92595 (11)	0.5586 (4)	0.0237 (6)	
C21	0.3663 (3)	0.94142 (13)	0.6653 (5)	0.0264 (8)	
C22	0.2489 (4)	0.94383 (14)	0.5780 (6)	0.0292 (8)	
H22	0.196291	0.954631	0.649971	0.035*	
C23	0.2074 (4)	0.93070 (15)	0.3871 (6)	0.0323 (8)	
H23	0.126916	0.932915	0.327424	0.039*	
C24	0.2845 (4)	0.91433 (15)	0.2842 (6)	0.0308 (8)	
H24	0.257430	0.904713	0.153547	0.037*	
C25	0.4001 (4)	0.91215 (14)	0.3729 (6)	0.0284 (8)	
H25	0.453469	0.900781	0.303152	0.034*	
O31	0.6345 (2)	0.86796 (9)	1.0842 (4)	0.0246 (5)	
N31	0.7388 (3)	0.87132 (12)	1.2112 (5)	0.0268 (7)	
C31	0.7804 (4)	0.91637 (15)	1.2867 (6)	0.0302 (8)	
C32	0.8873 (4)	0.91678 (17)	1.4195 (6)	0.0335 (9)	
H32	0.918314	0.948072	1.474374	0.040*	
C33	0.9489 (4)	0.87328 (17)	1.4731 (6)	0.0342 (9)	
H33	1.021778	0.874207	1.564265	0.041*	
C34	0.9030 (4)	0.82742 (16)	1.3916 (6)	0.0331 (9)	
H34	0.944526	0.796754	1.427124	0.040*	
C35	0.7986 (4)	0.82690 (15)	1.2613 (6)	0.0297 (8)	
H35	0.767106	0.795842	1.204877	0.036*	
C36	0.7088 (4)	0.96175 (16)	1.2225 (7)	0.0365 (9)	
H36A	0.637607	0.959791	1.267447	0.055*	
H36B	0.688837	0.963430	1.082058	0.055*	
H36C	0.752416	0.992135	1.275386	0.055*	
C16	0.6740 (4)	0.72348 (16)	0.8382 (6)	0.0325 (8)	
H16A	0.648259	0.746150	0.927280	0.049*	
H16B	0.755859	0.730027	0.843848	0.049*	
H16C	0.664650	0.688212	0.874079	0.049*	
C26	0.4185 (4)	0.95516 (14)	0.8691 (6)	0.0289 (8)	
H26A	0.480012	0.980250	0.875055	0.043*	
H26B	0.451361	0.924805	0.940647	0.043*	
H26C	0.358631	0.969462	0.925716	0.043*	

### Atomic displacement parameters (Å^2^)

**Table:**

	*U*^11^	*U*^22^	*U*^33^	*U*^12^	*U*^13^	*U*^23^
Co1	0.0208 (3)	0.0204 (2)	0.0194 (3)	−0.0007 (2)	0.0083 (2)	−0.0004 (2)
N1	0.0219 (14)	0.0255 (13)	0.0247 (14)	−0.0035 (11)	0.0089 (12)	−0.0014 (11)
C1	0.0264 (17)	0.0220 (14)	0.0193 (16)	−0.0011 (13)	0.0047 (13)	0.0005 (12)
S1	0.0238 (4)	0.0396 (5)	0.0308 (5)	−0.0034 (3)	0.0124 (4)	0.0036 (4)
N2	0.0219 (14)	0.0265 (14)	0.0255 (15)	−0.0026 (11)	0.0079 (12)	−0.0007 (11)
C2	0.0276 (18)	0.0223 (15)	0.0213 (16)	0.0036 (12)	0.0088 (14)	0.0012 (11)
S2	0.0256 (4)	0.0383 (5)	0.0296 (5)	−0.0083 (4)	0.0060 (4)	−0.0015 (4)
O11	0.0232 (12)	0.0260 (11)	0.0377 (15)	−0.0062 (9)	0.0148 (11)	−0.0096 (10)
N11	0.0217 (14)	0.0237 (13)	0.0336 (16)	−0.0020 (11)	0.0143 (12)	−0.0075 (12)
C11	0.0230 (16)	0.0278 (16)	0.0301 (19)	−0.0001 (13)	0.0116 (15)	−0.0012 (14)
C12	0.0269 (17)	0.0265 (16)	0.0327 (19)	−0.0009 (14)	0.0175 (15)	−0.0027 (14)
C13	0.0246 (17)	0.0336 (18)	0.0306 (19)	−0.0011 (14)	0.0114 (15)	−0.0089 (15)
C14	0.0259 (18)	0.0359 (19)	0.0277 (18)	0.0032 (15)	0.0104 (15)	0.0000 (14)
C15	0.0310 (18)	0.0277 (17)	0.0312 (19)	0.0055 (14)	0.0164 (15)	0.0025 (14)
O21	0.0229 (12)	0.0274 (12)	0.0307 (13)	−0.0003 (9)	0.0077 (10)	0.0069 (10)
N21	0.0233 (15)	0.0228 (13)	0.0252 (15)	0.0016 (11)	0.0060 (12)	0.0055 (11)
C21	0.0318 (19)	0.0220 (15)	0.0267 (18)	0.0004 (13)	0.0096 (15)	0.0024 (12)
C22	0.031 (2)	0.0309 (17)	0.0289 (19)	0.0027 (14)	0.0135 (16)	0.0053 (14)
C23	0.0272 (19)	0.0361 (19)	0.032 (2)	−0.0040 (15)	0.0055 (16)	0.0051 (16)
C24	0.035 (2)	0.0325 (18)	0.0242 (17)	−0.0044 (15)	0.0068 (16)	0.0025 (14)
C25	0.0323 (19)	0.0290 (17)	0.0260 (18)	0.0024 (15)	0.0108 (15)	0.0033 (14)
O31	0.0224 (12)	0.0322 (13)	0.0203 (12)	−0.0040 (9)	0.0071 (10)	−0.0003 (9)
N31	0.0277 (16)	0.0349 (16)	0.0206 (14)	−0.0067 (12)	0.0117 (13)	−0.0007 (11)
C31	0.036 (2)	0.0322 (18)	0.0263 (18)	−0.0067 (15)	0.0155 (16)	−0.0013 (14)
C32	0.031 (2)	0.048 (2)	0.0242 (18)	−0.0095 (17)	0.0117 (15)	−0.0030 (16)
C33	0.029 (2)	0.051 (2)	0.0251 (19)	−0.0066 (16)	0.0116 (16)	0.0017 (16)
C34	0.035 (2)	0.0381 (19)	0.029 (2)	0.0036 (17)	0.0148 (17)	0.0053 (15)
C35	0.0329 (19)	0.0331 (18)	0.0271 (18)	−0.0052 (15)	0.0149 (15)	−0.0006 (14)
C36	0.037 (2)	0.0340 (19)	0.040 (2)	−0.0022 (16)	0.0138 (18)	−0.0041 (17)
C16	0.031 (2)	0.0377 (19)	0.030 (2)	−0.0020 (15)	0.0085 (16)	0.0011 (15)
C26	0.0312 (19)	0.0300 (17)	0.0270 (18)	0.0031 (15)	0.0103 (15)	−0.0015 (14)

### Geometric parameters (Å, º)

**Table:**

Co1—N1	2.086 (3)	C22—C23	1.384 (6)
Co1—N2	2.051 (3)	C23—H23	0.9500
Co1—O11	1.992 (3)	C23—C24	1.382 (7)
Co1—O21	2.016 (2)	C24—H24	0.9500
Co1—O31	2.037 (3)	C24—C25	1.368 (6)
N1—C1	1.162 (5)	C25—H25	0.9500
C1—S1	1.637 (4)	O31—N31	1.351 (4)
N2—C2	1.158 (5)	N31—C31	1.350 (5)
C2—S2	1.639 (4)	N31—C35	1.373 (5)
O11—N11	1.335 (4)	C31—C32	1.390 (6)
N11—C11	1.350 (5)	C31—C36	1.477 (6)
N11—C15	1.358 (5)	C32—H32	0.9500
C11—C12	1.397 (5)	C32—C33	1.366 (7)
C11—C16	1.481 (5)	C33—H33	0.9500
C12—H12	0.9500	C33—C34	1.396 (6)
C12—C13	1.370 (6)	C34—H34	0.9500
C13—H13	0.9500	C34—C35	1.359 (6)
C13—C14	1.385 (6)	C35—H35	0.9500
C14—H14	0.9500	C36—H36A	0.9800
C14—C15	1.367 (6)	C36—H36B	0.9800
C15—H15	0.9500	C36—H36C	0.9800
O21—N21	1.344 (4)	C16—H16A	0.9800
N21—C21	1.358 (5)	C16—H16B	0.9800
N21—C25	1.352 (5)	C16—H16C	0.9800
C21—C22	1.384 (6)	C26—H26A	0.9800
C21—C26	1.489 (5)	C26—H26B	0.9800
C22—H22	0.9500	C26—H26C	0.9800
			
N2—Co1—N1	176.35 (13)	C24—C23—H23	120.4
O11—Co1—N1	91.87 (12)	C23—C24—H24	120.4
O11—Co1—N2	88.87 (12)	C25—C24—C23	119.2 (4)
O11—Co1—O21	114.88 (12)	C25—C24—H24	120.4
O11—Co1—O31	126.83 (11)	N21—C25—C24	120.8 (4)
O21—Co1—N1	91.23 (11)	N21—C25—H25	119.6
O21—Co1—N2	91.70 (12)	C24—C25—H25	119.6
O21—Co1—O31	118.20 (11)	N31—O31—Co1	117.8 (2)
O31—Co1—N1	84.17 (12)	O31—N31—C35	116.9 (3)
O31—Co1—N2	92.52 (12)	C31—N31—O31	121.0 (3)
C1—N1—Co1	155.7 (3)	C31—N31—C35	122.2 (4)
N1—C1—S1	178.6 (3)	N31—C31—C32	117.8 (4)
C2—N2—Co1	177.5 (3)	N31—C31—C36	117.6 (4)
N2—C2—S2	179.4 (4)	C32—C31—C36	124.6 (4)
N11—O11—Co1	119.1 (2)	C31—C32—H32	119.2
O11—N11—C11	120.5 (3)	C33—C32—C31	121.6 (4)
O11—N11—C15	117.7 (3)	C33—C32—H32	119.2
C11—N11—C15	121.8 (3)	C32—C33—H33	120.5
N11—C11—C12	118.1 (3)	C32—C33—C34	118.9 (4)
N11—C11—C16	117.4 (3)	C34—C33—H33	120.5
C12—C11—C16	124.5 (4)	C33—C34—H34	120.2
C11—C12—H12	119.6	C35—C34—C33	119.6 (4)
C13—C12—C11	120.8 (4)	C35—C34—H34	120.2
C13—C12—H12	119.6	N31—C35—H35	120.0
C12—C13—H13	120.3	C34—C35—N31	119.9 (4)
C12—C13—C14	119.4 (4)	C34—C35—H35	120.0
C14—C13—H13	120.3	C31—C36—H36A	109.5
C13—C14—H14	120.4	C31—C36—H36B	109.5
C15—C14—C13	119.3 (4)	C31—C36—H36C	109.5
C15—C14—H14	120.4	H36A—C36—H36B	109.5
N11—C15—C14	120.6 (3)	H36A—C36—H36C	109.5
N11—C15—H15	119.7	H36B—C36—H36C	109.5
C14—C15—H15	119.7	C11—C16—H16A	109.5
N21—O21—Co1	120.8 (2)	C11—C16—H16B	109.5
O21—N21—C21	119.6 (3)	C11—C16—H16C	109.5
O21—N21—C25	118.7 (3)	H16A—C16—H16B	109.5
C25—N21—C21	121.7 (3)	H16A—C16—H16C	109.5
N21—C21—C22	118.3 (3)	H16B—C16—H16C	109.5
N21—C21—C26	117.4 (3)	C21—C26—H26A	109.5
C22—C21—C26	124.4 (4)	C21—C26—H26B	109.5
C21—C22—H22	119.6	C21—C26—H26C	109.5
C23—C22—C21	120.8 (4)	H26A—C26—H26B	109.5
C23—C22—H22	119.6	H26A—C26—H26C	109.5
C22—C23—H23	120.4	H26B—C26—H26C	109.5
C24—C23—C22	119.2 (4)		

### Hydrogen-bond geometry (Å, º)

**Table:**

*D*—H···*A*	*D*—H	H···*A*	*D*···*A*	*D*—H···*A*
C14—H14···S1^i^	0.95	2.95	3.868 (4)	163
C15—H15···O31^i^	0.95	2.47	3.296 (5)	145
C32—H32···S2^ii^	0.95	3.02	3.873 (4)	150
C26—H26*B*···O31	0.98	2.64	3.512 (5)	149

Symmetry codes: (i) *x*, *y*, *z*−1; (ii) *x*, −*y*+2, *z*+1/2.
