# Supplementary figures and images for: Synthesis, crystal structure and properties of the trigonal–bipyramidal complex tris­(2-methyl­pyridine N-oxide-κO)bis­(thio­cyanato-κN)cobalt(II)
Source: Acta Crystallogr E Crystallogr Commun. 2024 Apr 11;80(Pt 5):463–7. doi: 10.1107/S2056989024003050 (PMC11074571; doi:10.1107/S2056989024003050)

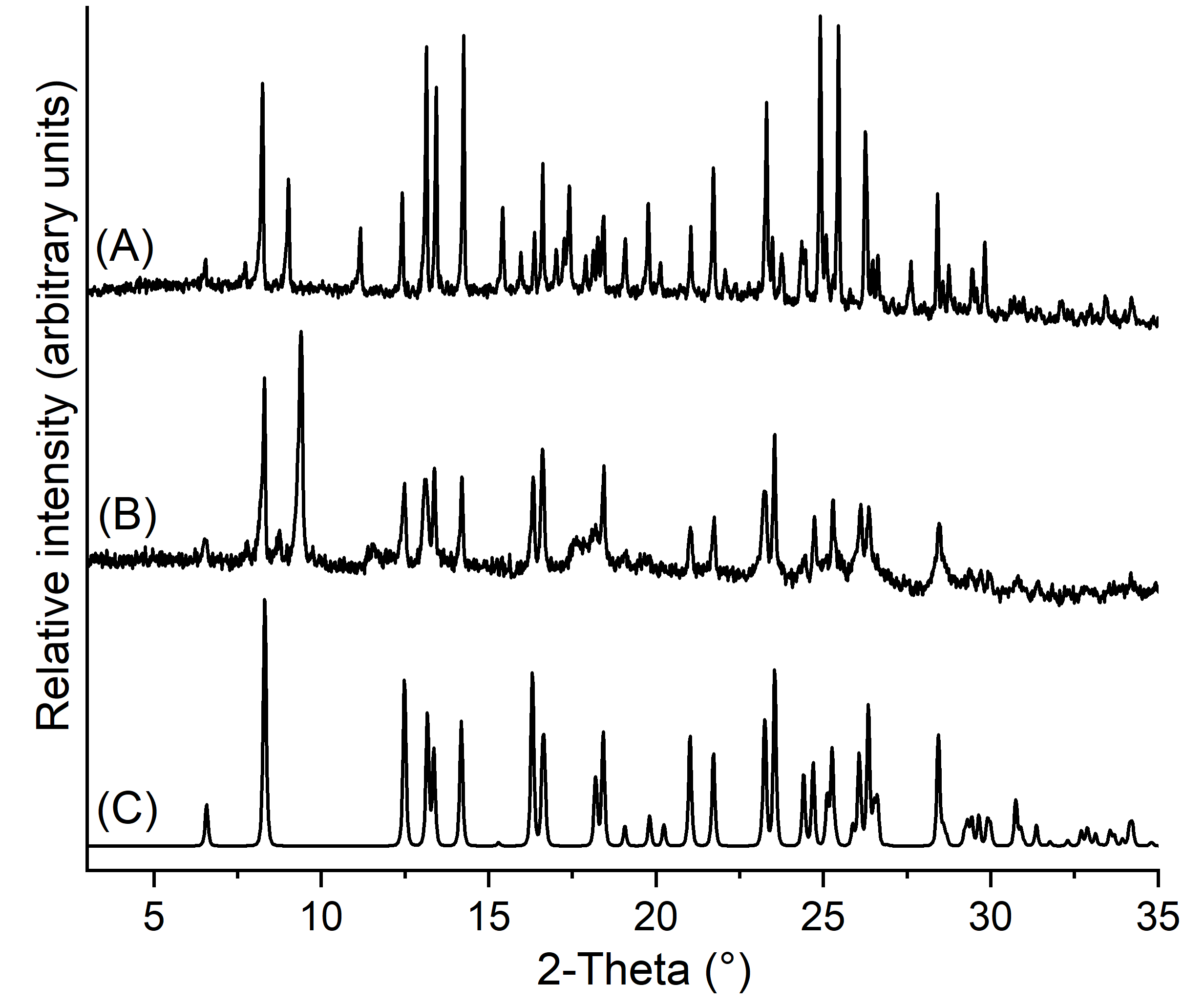

Supplement: Supplementary file 3 [file e-80-00463-sup3.png]

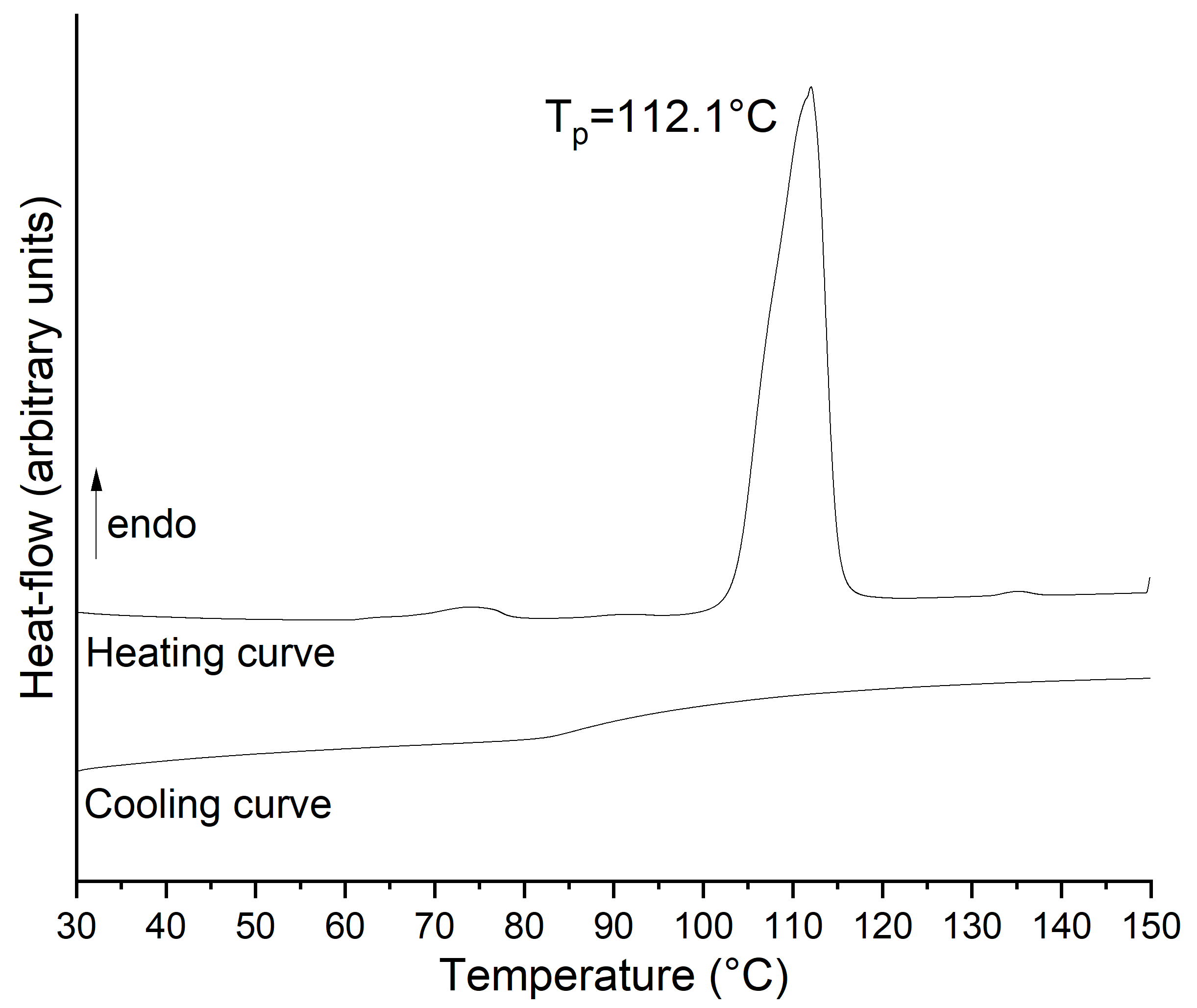

Supplement: Supplementary file 4 [file e-80-00463-sup4.png]

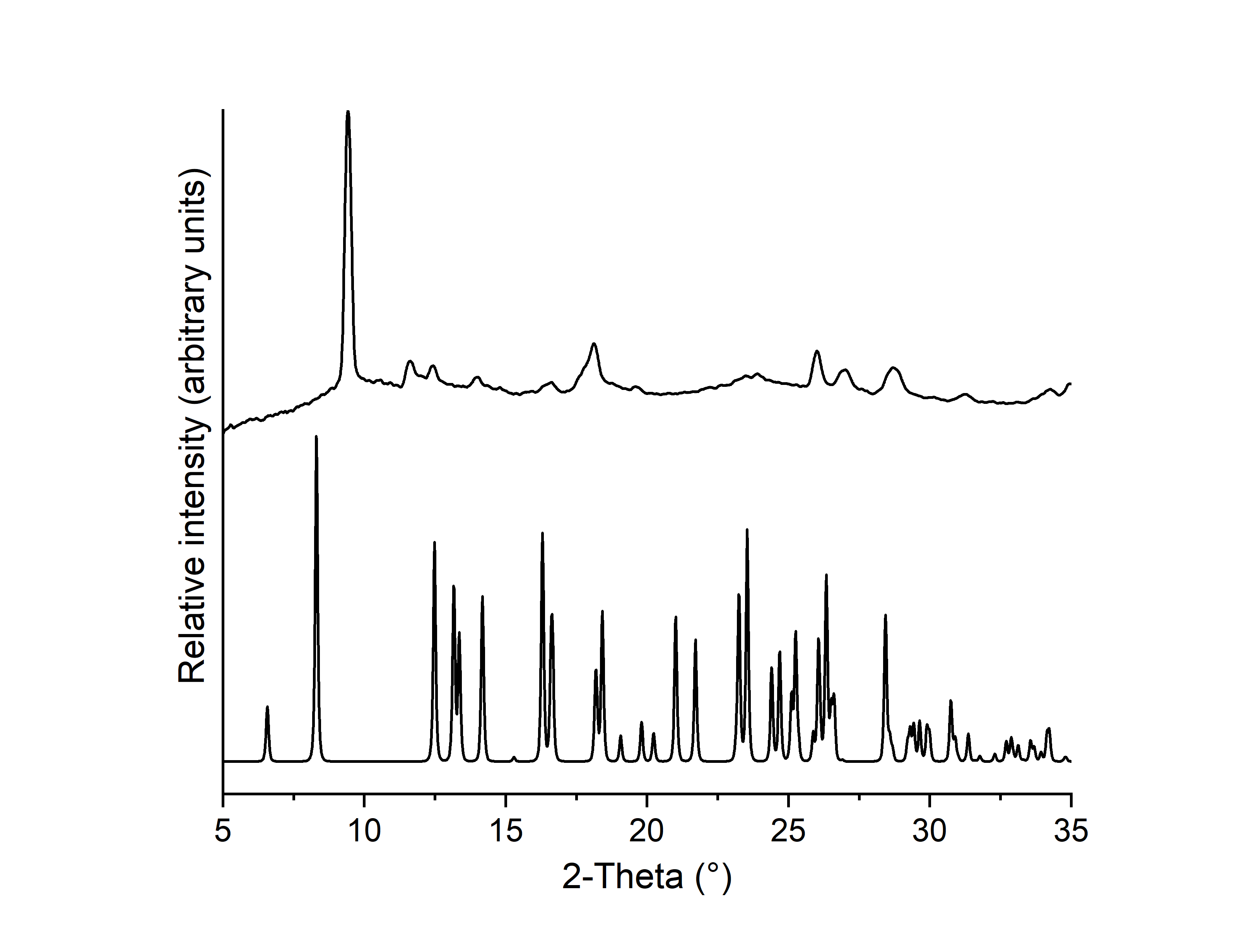

Supplement: Supplementary file 5 [file e-80-00463-sup5.png]

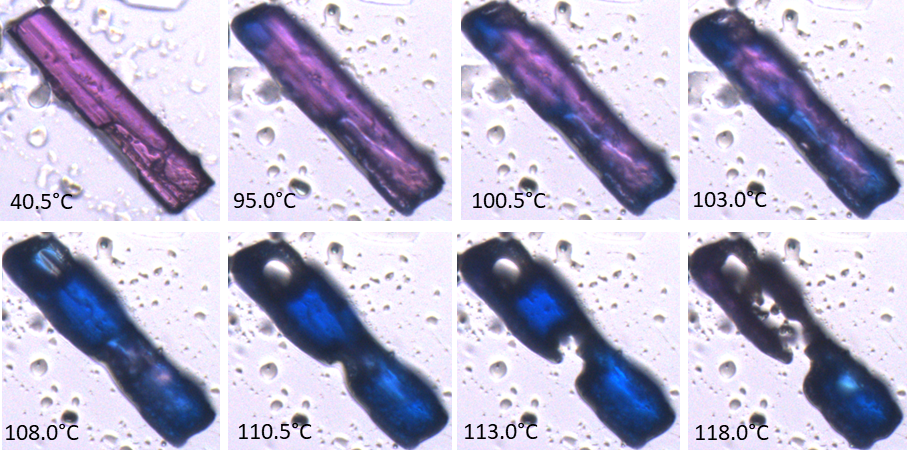

Supplement: Supplementary file 6 [file e-80-00463-sup6.png]
